# Bioinformatics Analysis Reveals Distinct Molecular Characteristics of Hepatitis B-Related Hepatocellular Carcinomas from Very Early to Advanced Barcelona Clinic Liver Cancer Stages

**DOI:** 10.1371/journal.pone.0158286

**Published:** 2016-07-25

**Authors:** Fan-Yun Kong, Xiao Wei, Kai Zhou, Wei Hu, Yan-Bo Kou, Hong-Juan You, Xiao-Mei Liu, Kui-Yang Zheng, Ren-Xian Tang

**Affiliations:** Department of Pathogenic Biology and Immunology, Laboratory of Infection and Immunity, Xuzhou Medical College, Xuzhou, Jiangsu, 221004, China; University of Cincinnati College of Medicine, UNITED STATES

## Abstract

Hepatocellular carcinoma (HCC)is the fifth most common malignancy associated with high mortality. One of the risk factors for HCC is chronic hepatitis B virus (HBV) infection. The treatment strategy for the disease is dependent on the stage of HCC, and the Barcelona clinic liver cancer (BCLC) staging system is used in most HCC cases. However, the molecular characteristics of HBV-related HCC in different BCLC stages are still unknown. Using GSE14520 microarray data from HBV-related HCC cases with BCLC stages from 0 (very early stage) to C (advanced stage) in the gene expression omnibus (GEO) database, differentially expressed genes (DEGs), including common DEGs and unique DEGs in different BCLC stages, were identified. These DEGs were located on different chromosomes. The molecular functions and biology pathways of DEGs were identified by gene ontology (GO) analysis and Kyoto Encyclopedia of Genes and Genomes (KEGG) pathway analysis, and the interactome networks of DEGs were constructed using the NetVenn online tool. The results revealed that both common DEGs and stage-specific DEGs were associated with various molecular functions and were involved in special biological pathways. In addition, several hub genes were found in the interactome networks of DEGs. The identified DEGs and hub genes promote our understanding of the molecular mechanisms underlying the development of HBV-related HCC through the different BCLC stages, and might be used as staging biomarkers or molecular targets for the treatment of HCC with HBV infection.

## Introduction

Hepatocellular carcinoma (HCC) is the fifth most common malignancy in adults and it is associated with high mortality. The incidence of HCC has been reported to differ widely in various regions. The highest incidence of HCC is found in developing regions such as Asia and Africa, and especially in China, which accounts for more than 50% of HCC cases around the world[1, 2]. The unequal distribution of HCC in different geographic areas suggests that a variety of genetic and environmental factors contribute to the development of this disease. In addition, epidemiological studies indicate that one of the important risk factors for HCC is chronic infection with hepatitis B virus (HBV); in China, there are more than 93 million HBV carriers[3]. Therefore, it is very important to identify the molecular mechanisms underlying the development of HBV-related HCC to identify potential therapeutic targets for HCC with HBV infection.

Currently, treatment strategies depend primarily on the stage of HCC, and the Barcelona clinic liver cancer (BCLC) staging system is used most for HCC patients[4]. For HCC patients with BCLC stage 0 (very early stage)or A (early stage), surgical resection, liver transplantation, and radiofrequency ablation are considered curative treatments. For patients with HCC in BCLC stage B(intermediate stage)or C (advanced stage), transarterial chemoembolization and sorafenib treatments are often recommended. In addition, if HCC patients are in BCLC stage D (end stage), the suggested treatment option is symptomatic therapy [5].Current treatment approaches are nonspecific, and the chemotherapy is highly toxic and associated with severe drug resistance[6]. A potential alternative approach for cancer treatment is developing new agents that modify cancer cell differentiation, termed “differentiation therapy”[7, 8]. This strategy is based on the understanding that specific subtypes of many tumors are related to characteristic alterations in normal programs of cell differentiation and growth control that could be corrected with appropriate treatments. Therefore, it is very important to elucidate the molecular mechanisms of differentiation and growth of HCC at different BCLC stages.

The differentially expressed genes (DEGs) and associated molecular abnormalities of HCC, especially HBV-related HCC, have been explored by many different groups [9–14]. However, to our knowledge, the reported studies mainly focused on the identification of distinct gene expression signatures and their usefulness as molecular markers in the prediction of clinical outcomes such as survival, metastasis, and recurrence in patients with HCC [12–14]. The relationship between genetic factors and related mechanisms at each BCLC stage of HBV-related HCC has not been well elucidated. In order to better understand the molecular characteristics of HBV-related HCC in different BCLC stages, DEGs, their associated biological function and pathways, and their interactome networks were analyzed with a gene expression microarray of HBV-related HCC cases from the GEO database. Our investigation contributes to identifying potential biomarkers and therapeutic targets of HBV-related HCC in different BCLC stages.

## Materials and Methods

### Data source

The microarray data in the present study was retrieved and downloaded from the NCBI gene expression omnibus (GEO) database (http://www.ncbi.nlm.nih.gov/geo/) using keywords, including (“hepatocellular carcinoma” or “HCC”) and (“hepatitis B” or “HBV”). After screening the entries for information on HCC BCLC stages, we chose the microarray dataset GSE14520 [15, 16] as our subject for analysis, and the details of microarray processing were reported by Roessler S et al[15]. Samples in GSE14520 were primarily analyzed with Affymetrix HT Human Genome U133A Array (GPL3921 platform); only 22 samples were analyzed by Affymetrix Human Genome U133A 2.0 Array (GPL571 platform). In order to avoid the effect of different detection platforms on the analysis, we removed the samples analyzed with the GPL571 platform from our analysis. In addition, the HCC samples without HBV infection or HBV-related HCC samples without BCLC stage information were excluded from the present study. Finally, 220 non-HCC cases, 20 HBV-related HCC cases in BCLC stage 0, 143 HBV-related HCC cases in BCLC stage A, 22 HBV-related HCC cases in BCLC stage B, and 27 HBV-related HCC cases in BCLC stage C were analyzed in the present study (S1 Table).

### Data processing and identification of differential gene expression

After extracted from GSE14520, the raw data files were performed with Affymetrix Expression Console and Affymetrix Transcriptome Analysis Console v3.0, which were easy-to-use software package openly available to all Affymetrix expression array users. In addition, the researchers could normalize, perform data quality control, and summarize data from thousands of samples as well as tens of thousands of genes, by using these two software packages (http://www.affymetrix.com/estore/browse/level_seven_software_productsonly.jsp?productId=131414#11). The detailed analysis was according to the manufacturer's protocol. Briefly, using Expression Console, probe signal values were converted to log_2_ values, genes annotated by the probes were analyzed according to Affymetrix HT Human Genome U133A array annotation files, and data were normalized using the robust multi-array average (RMA) algorithm. In addition, gene expression profiles of HBV-related HCC in different BCLC stages were compared to those of non-HCC to identify DEGs. Furthermore, we performed a statistical analysis by one-way ANOVA to identify significant DEGs (fold change ≥ 2.0, and *P* value < 0.05) using Transcriptome Analysis Console.

### Gene function and pathway enrichment analysis

The common DEGs and stage-specific DEGs in HBV-related HCC were identified and then subjected to gene ontology (GO) analysis to determine clusters of DEGs with enriched molecular functions. Kyoto Encyclopedia of Genes and Genomes (KEGG) pathway analysis was used to identify relevant biological pathways of clustered DEGs. In addition, we used the database for annotation, visualization and integrated discovery (DAVID) v6.7 online tool (http://david.abcc.ncifcrf.gov) [17, 18] to functionally annotate input genes, classify gene functions, and identify gene conversions, and to perform the GO and KEGG pathway analyses. A *P* value of < 0.05 was considered significant.

### Construction of the interactome network of DEGs

In order to construct the interactome network of DEGs, the common DEGs and stage-specific DEGs in HBV-related HCC were uploaded to an integrated network analysis web platform, NetVenn (http://wheat.pw.usda.gov/NetVenn) [19]. With this program, we constructed an interactome network based on protein–protein interaction (PPI) data that is publically available for several species, and visualized gene relationships with online graphic tool.

## Results

### Patient characteristics and associated factors with clinical outcomes in HBV-related HCC

As shown in Table 1,212 HBV-realted HCC patients, including 183 males and 29 females with a mean age of 50.6 years were enrolled in the study. There were 88 (42%) patients with an alanine aminotransferase (ALT) level more than 50 U/L, 94 (44%) patients with an alpha-fetoprotein (AFP) level over 300 ng/ml, 74 (35%) patients with a main tumour size over 5 cm, 45 (21%) patients with multimodular tumours, and 195 (92%) patients had underlying cirrhosis. Of these HBV-realted HCC patients, most patients (143, 67%) were diagnosed with BCLC staging A. We next investigated the clinical factors associated with outcomes such as survival and recurrence in HBV-related HCC by using Cox regression analysis. The results showed that, among these clinical factors, BCLC staging was not only associated with poor survival but also related to recurrence of the disease (Tables 2 and 3).Cirrhosis was found to be associated with poor survival of the disease (Table 2). In addition, multinodular was identified to contribute to recurrence of HBV-related HCC patients (Table 3).

**Table 1:** Baseline characteristics of HBV-related HCC patients.

Category	n / mean±SD
Gender (male/female)	183/29
Age (years)	50.6±10.6
ALT (>50/<50, U/L)	88/124
AFP (>300/<300/NA, ng/ml)	94/115/3
Main tumour size (>5/<5/NA, cm)	74/137/1
Multinodular (yes/no)	45/167
Cirrhosis (yes/no)	195/17
BCLC staging (0/A/B/C)	20/143/22/27

NA, not available.

**Table 2:** Cox regression analysis of risk factors associated with survival of HBV-related HCC patients.

Covariates	Hazard ratio	P value	95% CI
Gender	1.29	0.50	0.61–2.74
Age	0.99	0.26	0.97–1.01
ALT	1.34	0.22	0.84–2.15
Main tumor size	1.14	0.63	0.68–1.90
Multinodular	0.66	0.14	0.37–1.15
Cirrhosis	4.34	0.04	1.04–18.12
BCLC staging	2.56	<0.01	1.86–3.52
AFP	0.92	0.70	0.60–1.41

**Table 3:** Cox regression analysis of risk factors associated with recurrence of HBV-related HCC patients.

Covariates	Hazard ratio	P value	95% CI
Gender	1.81	0.08	0.94–3.52
Age	1.00	0.89	0.98–1.02
ALT	1.01	0.95	0.68–1.51
Main tumor size	1.32	0.22	0.85–2.04
Multinodular	0.55	0.03	0.32–0.94
Cirrhosis	2.33	0.07	0.93–5.85
BCLC staging	2.19	<0.01	1.65–2.92
AFP	1.01	0.97	0.70–1.44

### The identification of common DEGs and stage-specific DEGs in HBV-related HCC

**Fig 1:**
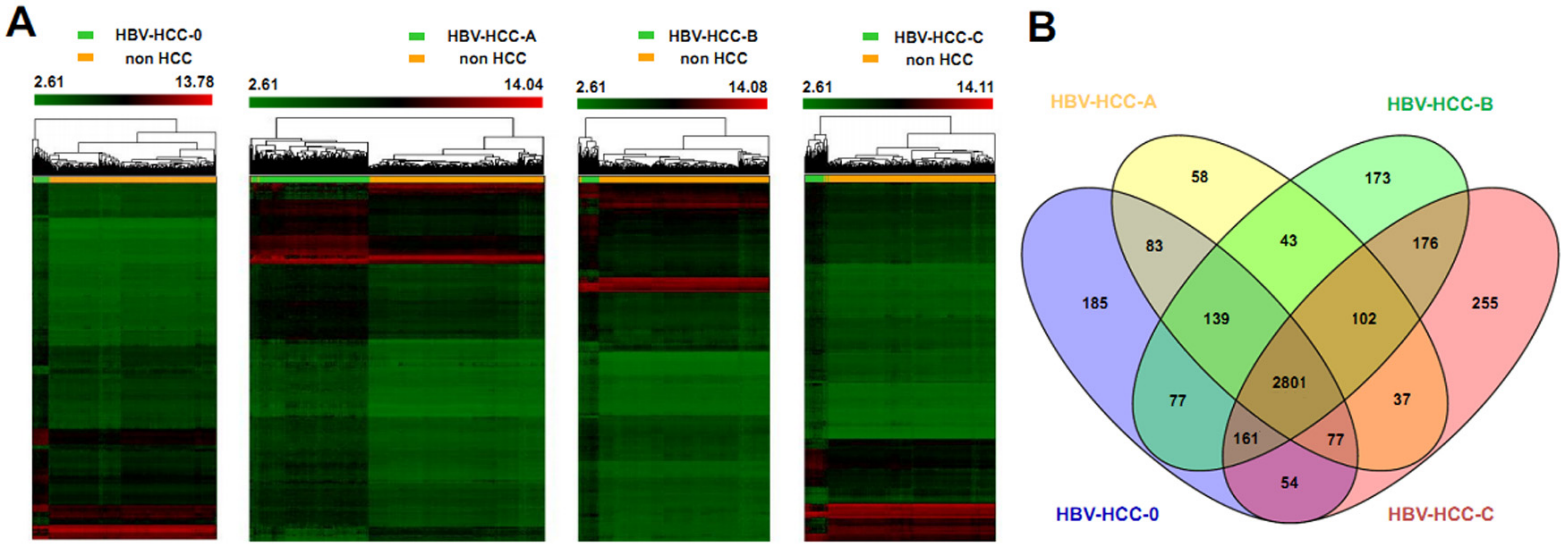
The identification of total DEGs, common DEGs, and stage-specific DEGs in HBV-related HCC. (A) Heatmap showing the differential gene expression profiles of HBV-related HCC cases with different BCLC stages compared to the non-HCC group. (B) The identification of common DEGs and stage-specific DEGs by Venn diagram. HBV-HCC-0 represents the HBV-related HCC cases with BCLC stage 0, HBV-HCC-A represents the HBV-related HCC cases with BCLC stage A, HBV-HCC-B represents the HBV-related HCC cases with BCLC stage B, and HBV-HCC-C represents the HBV-related HCC cases with BCLC stage C.

In the gene expression profiling analysis, we compared HBV-related HCC groups in different BCLC stages and the non-HCC group. A ≥2.0-fold change and a *P* valve <0.05 were considered significant. As shown in Fig 1A, 3581 DEGs in HBV-related HCC BCLC stage 0 (3212 genes upregulated and 269 genes downregulated,S2 Table), 3344 DEGs in HBV-related HCC BCLC stage A (3024 genes upregulated and 320 genes downregulated, S2 Table), 3676 DEGs in HBV-related HCC BCLC stage B (3237 genes upregulated and 439 genes downregulated, S2 Table), and 3667 DEGs in HBV-related HCC BCLC stage C (3142 genes upregulated and 525 genes downregulated, S2 Table) were identified, relative to non-HCC controls. In addition, we analyzed common DEGs and stage-specific DEGs in HBV-related HCC with BCLC stages from 0 to C with a Venn diagram using the Venny 2.0 online tool (Oliveros, 2007) (http://bioinfogp.cnb.csic.es/tools/venny/index.html). As shown in Fig 1B, we found that 2801 common DEGs existed in BCLC stages from 0 to C. Detailed gene information can be found in S3 Table. In addition, 185 unique DEGs were found in BCLC stage 0, 58 unique DEGs were found in BCLC stage A, 173 unique DEGs were found in BCLC stage B, and 255 unique DEGs were found in BCLC stage C. Detail on stage-specific DEGs can be found in S3 Table.

### The chromosome (chr) distribution of common DEGs and stage-specific DEGs in HBV-related HCC

**Fig 2:**
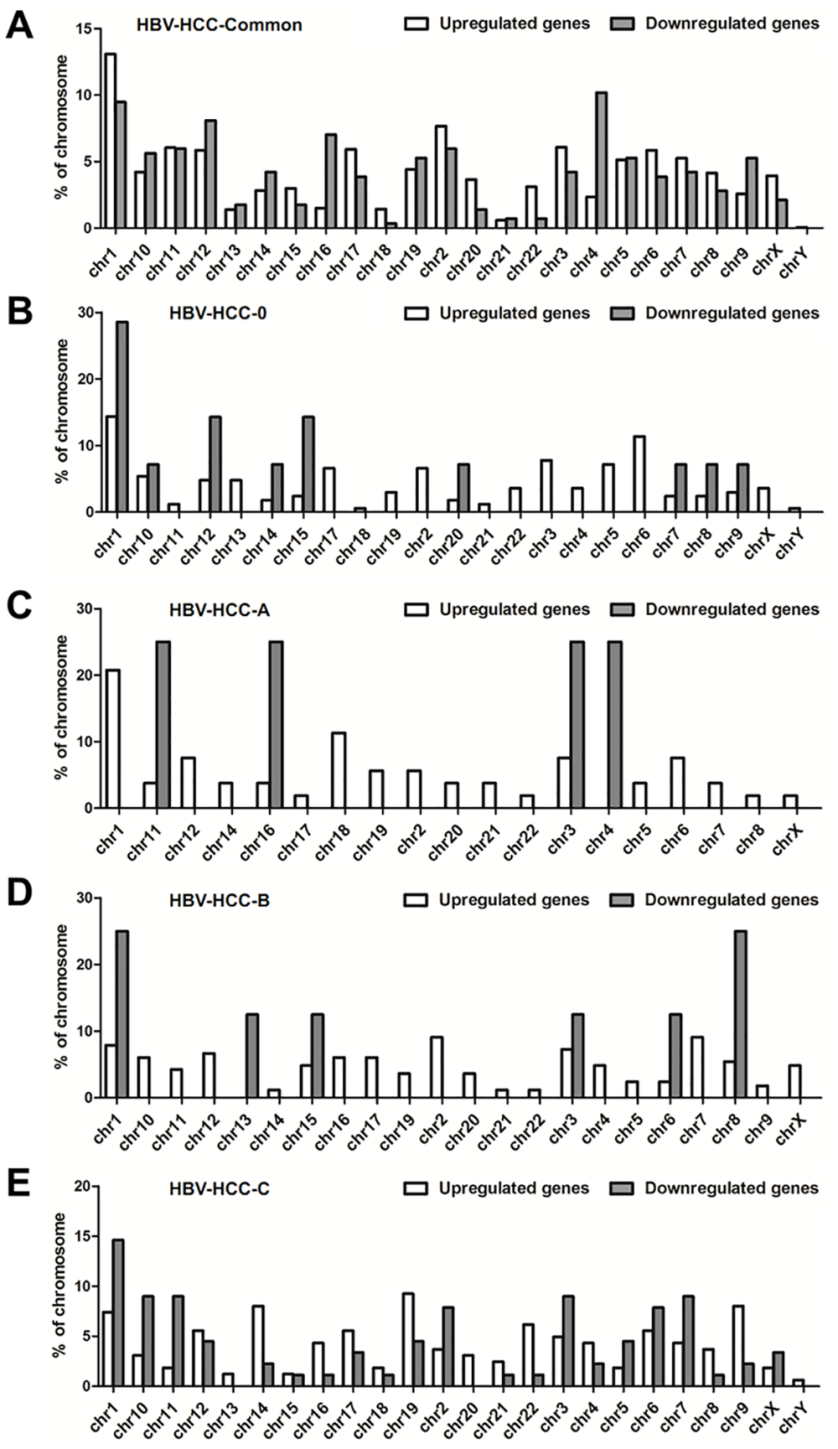
The distribution of common DEGs and stage-specific DEGs in HBV-related HCC in human chromosomes. (A) The locations of common DEGs on human chromosomes. (B) The chromosome distribution of unique DEGs in BCLC stage 0. (C) The chromosome locations of unique DEGs in BCLC stage A. (D) The chromosome distribution of unique DEGs in BCLC stage B. (E) The chromosome location of unique DEGs in BCLC stage C.

We next investigated the chromosomal location of common DEGs and stage-specific DEGs in HBV-related HCC groups in different BCLC stages. As shown in Fig 2A, we observed that the upregulated and downregulated common DEGs were widely distributed from chr 1 to chr X. We found that a relatively high number of upregulated common DEGs was distributed in chr 1 compared to other chromosomes, while relatively more downregulated common DEGs were found in chr4. In addition, we determined the distribution of unique DEGs in different BCLC stages of HBV-related HCC. As shown in Fig 2B–2E, both upregulated and downregulated unique DEGs in BCLC stage 0 were located mainly on chr 1. The upregulated unique DEGs in BCLC stage A were mainly distributed in chr 1, but the downregulated unique DEGs in BCLC stage A were located on chr 11, chr 16, chr3, and chr 4. For the unique DEGs in BCLC stage B, the upregulated genes were mainly found in chr 2, and the downregulated genes were located on chr 1 and chr 8. In BCLC stage C, the upregulated unique DEGs were mainly distributed in chr19, and the location of the downregulated unique DEGs were mainly on chr 1.

### The molecular function and pathway enrichment analysis of common DEGs and stage-specific DEGs in HBV-related HCC

**Fig 3:**
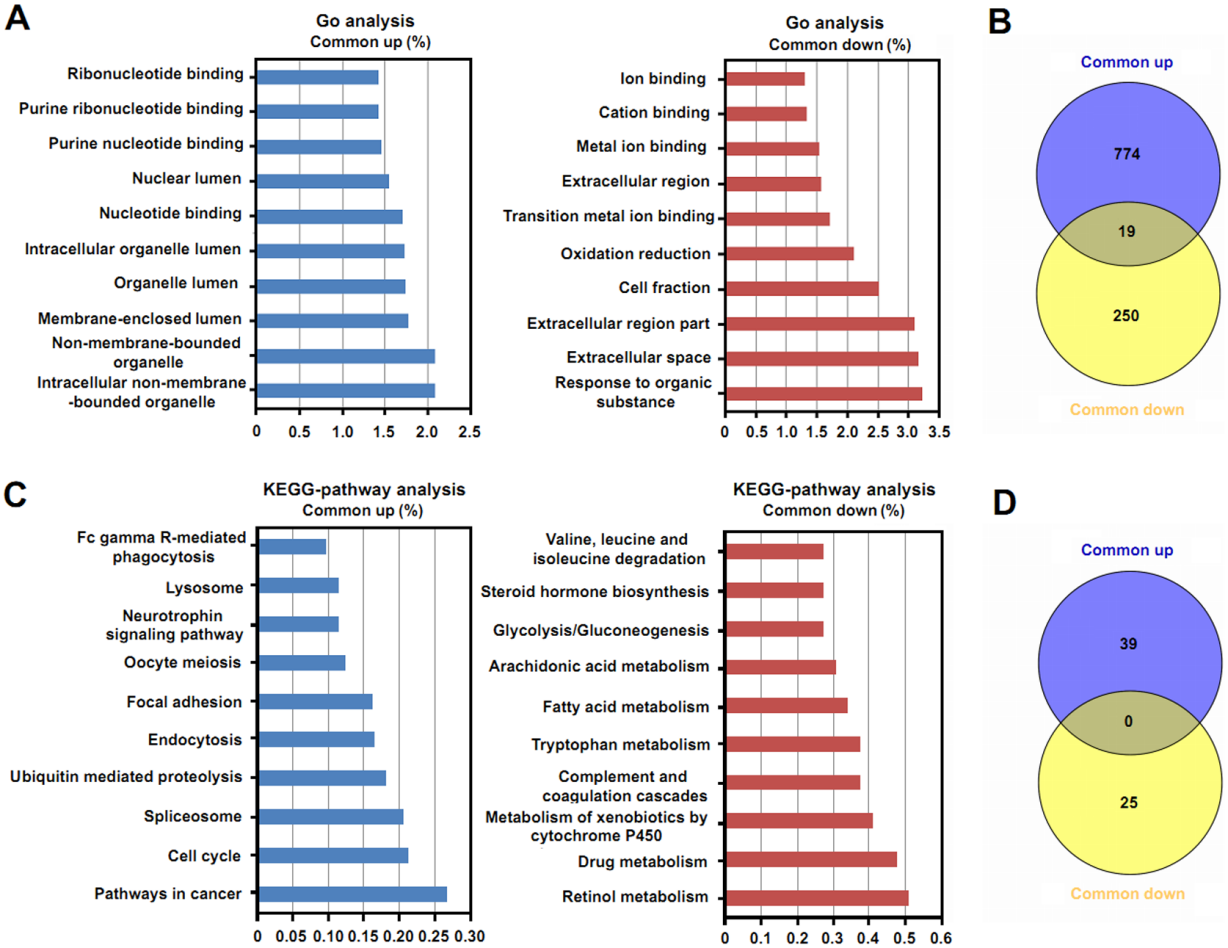
GO and KEGG analyses of common DEGs in HBV-related HCC. (A) The top 10 GO terms of upregulated and downregulated common DEGs. (B) The comparison of GO terms between upregulated DEGs and downregulated DEGs. (C) The top 10 KEGG pathways of upregulated and downregulated common DEGs. (D) The comparisonof KEGG pathways between upregulated DEGs and downregulated DEGs. “Common up” represents upregulated common DEGs;“Common down” represents downregulated common DEGs.

In order to investigate the molecular function and biology pathways of the common DEGs and stage-specific DEGs, GO and KEGG analyses were performed using the DAVID online tool. As for the common DEGs, multiple enriched GO terms and KEGG pathways were obtained (S4 and S5 Tables).The top 10 enriched GO terms and KEGG pathways of the upregulated and downregulated DEGs, according to the percentage of genes, were selected and are shown in Fig 3. Fig 3A indicates that the main enriched GO terms of upregulated DEGs were associated with biological processes involving non-membrane-bound organelles, binding, and lumens, and the main enriched GO terms of downregulated DEGs were related to binding and extracellular regions. Enriched KEGG pathways of the upregulated and downregulated common DEGs are shown in Fig 3C. We observed that the upregulated DEGs participated in multiple biology pathways, while the downregulated common DEGs were mainly associated with biosynthesis and metabolism. We also compared the enriched GO terms and KEGG pathways between the upregulated and downregulated common DEGs, as shown in Fig 3B and 3D, and the number of identical GO terms between upregulated and downregulated common DEGs was small. We did not find any KEGG pathways in common between regulated and downregulated common DEGs.

**Fig 4:**
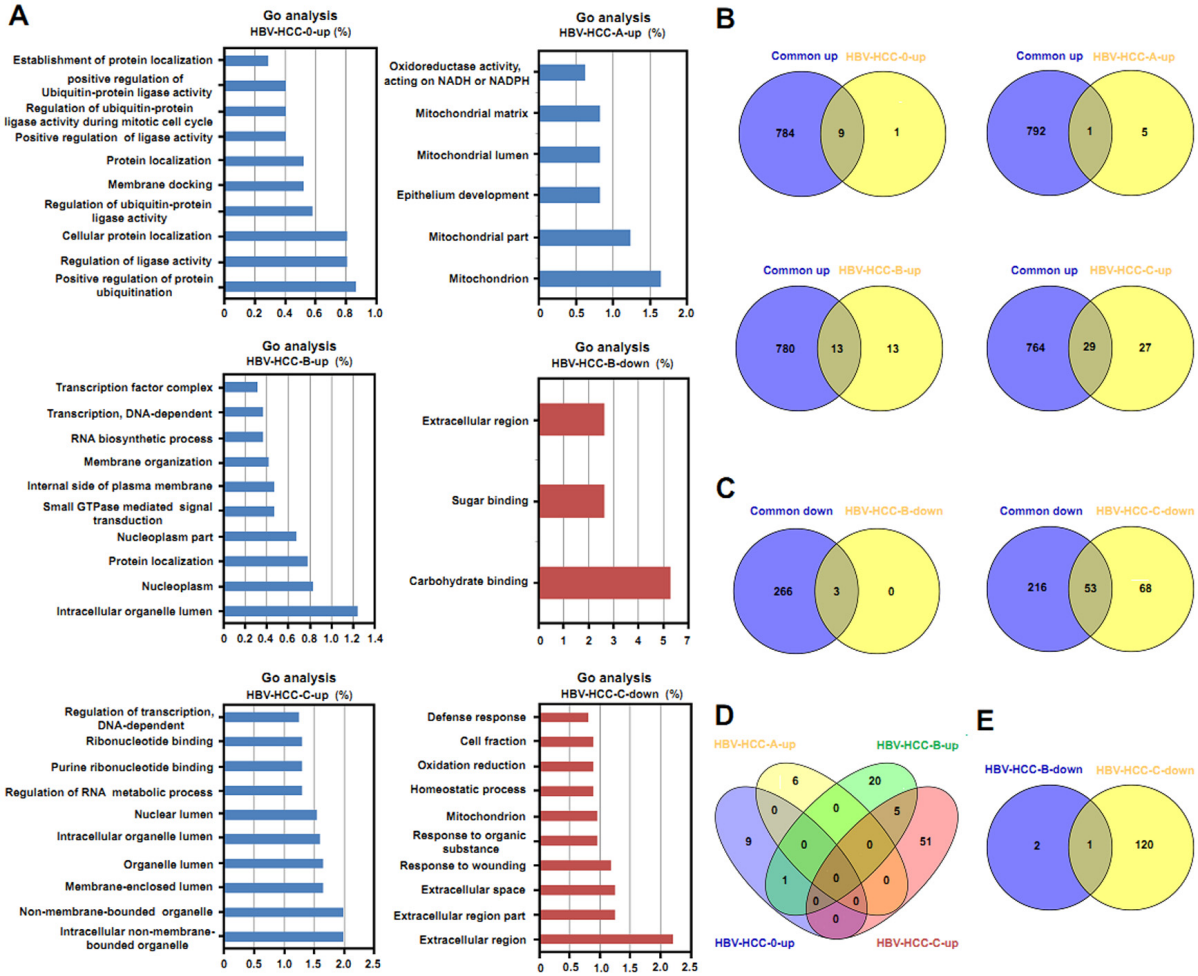
GO analysis of stage-specific DEGs in HBV-related HCC. (A) The top 10 GO terms of upregulated and downregulated stage-specific DEGs. (B) A comparison of GO terms of upregulated common DEGs and GO terms of upregulated stage-specific DEGs. (C) A comparison of GO terms of downregulated common DEGs and GO terms of downregulated stage-specific DEGs. (D) A comparison of GO terms of among upregulated stage-specific DEGs. (E) A comparison of GO terms among downregulated stage-specific DEGs. HBV-HCC-0-up means upregulated unique DEGs in BCLC stage 0, HBV-HCC-A-up means upregulated unique DEGs in BCLC stage A, HBV-HCC-B-up means upregulated unique DEGs in BCLC stage B, HBV-HCC-B-down means downregulated unique DEGs in BCLC stage B, HBV-HCC-C-up means upregulated unique DEGs in BCLC stage C, and HBV-HCC-C-down means downregulated unique DEGs in BCLC stage C.

We next performed GO and KEGG pathway analyses of unique DEGs in different BCLC stages of HBV-related HCC. As for GO terms, we did not find significantly enriched molecular functions in the downregulated unique DEGs in BCLC stages 0 and A. Fig 4A shows the enriched GO terms (at least the top 10 GO terms according to percentage of genes were selected) of unique DEGs from BCLC stage 0 to C in HBV-related HCC. We found that the molecular functions of upregulated and downregulated unique DEGs in different BCLC stages were distinct. Next, molecular functions of common DEGs and unique DEGs in different BCLC stages were compared. As shown in Fig 4B and 4C, we observed that common DEGs and unique DEGs in different BCLC stages were associated with relatively few shared molecular functions, and the main molecular functions of unique DEGs in different BCLC stages were distinct. GO terms of unique DEGs in different BCLC stages were also compared. As shown in Fig 4D and 4E, only one molecular function of upregulated unique DEGs was shared between BCLC stage 0 and BCLC stage B, and one molecular function of downregulated unique DEGs was shared between BCLC stage B and BCLC stage C.

**Fig 5:**
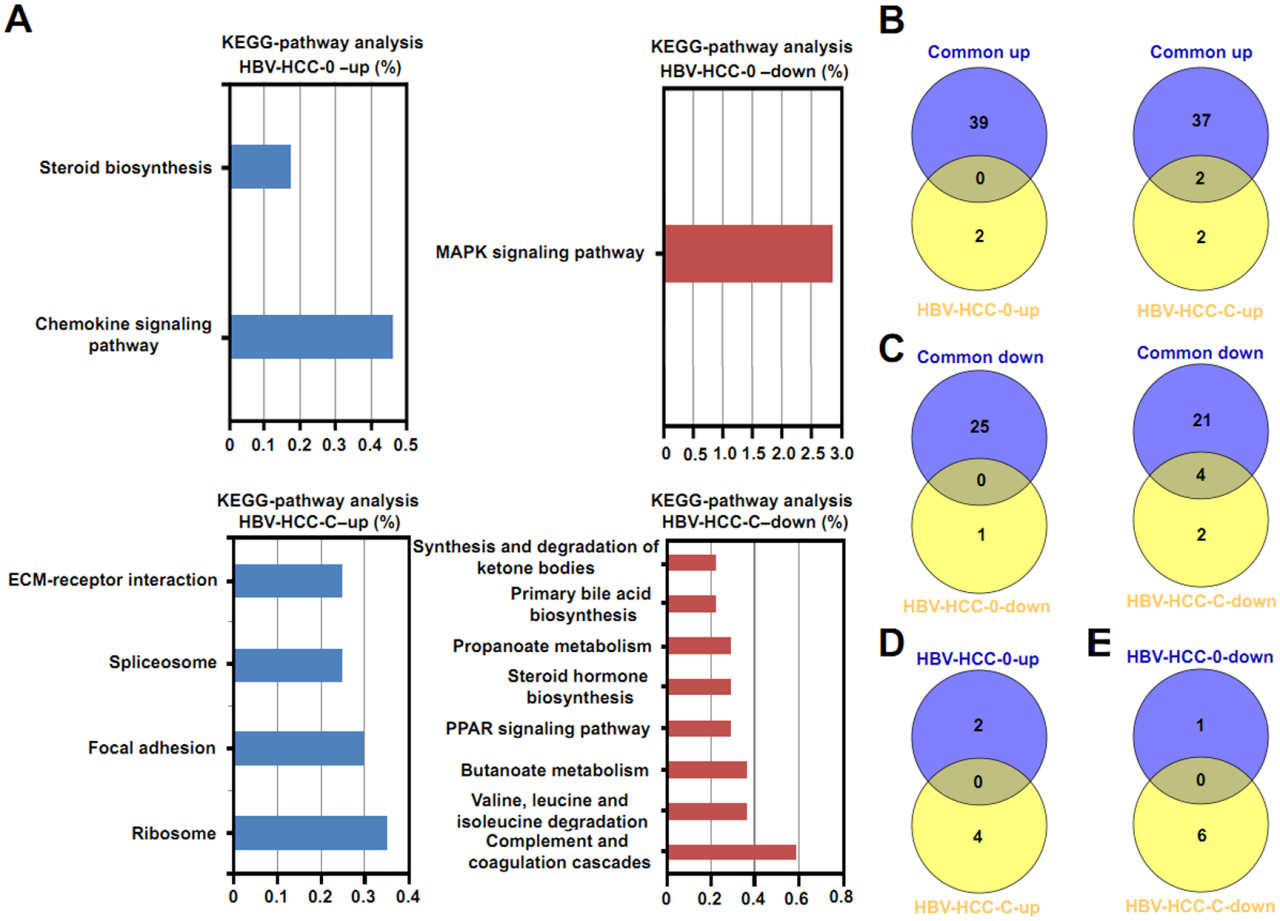
The KEGG pathway analysis of stage-specificDEGs in HBV-related HCC. (A) The top 10 KEGG pathways of upregulated and downregulated stage-specific DEGs. (B) A comparison of KEGG pathways of upregulated common DEGs with upregulated stage-specific DEGs. (C) A comparison of KEGG pathways of downregulated common DEGs with downregulated stage-specific DEGs. (D) A comparison of KEGG pathways among upregulated stage-specific DEGs. (E) A comparison of KEGG pathways among downregulated stage-specific DEGs.

As for the KEGG analysis, we only observed significantly enriched KEGG terms of upregulated DEGs and downregulated DEGs in BCLC stages 0 and C (Fig 5A).We compared the KEGG pathways between common DEGs and unique DEGs in stages 0 and C. The results showed that there were no shared KEGG pathways between the common DEGs and unique DEGs in BCLC stage 0. Only two KEGG pathways were shared between upregulated common DEGs and upregulated unique DEGs in BCLC stage C, and four KEGG pathways shared between downregulated common DEGs and downregulated unique DEGs in BCLC stage C. In addition, we compared the KEGG pathways of the unique DEGs between BCLC stage 0 and C, but we did not find any KEGG pathways of unique genes shared between BCLC stage 0 and C.

### The interactome networks of common DEGs and stage-specific DEGs in HBV-related HCC

**Fig 6:**
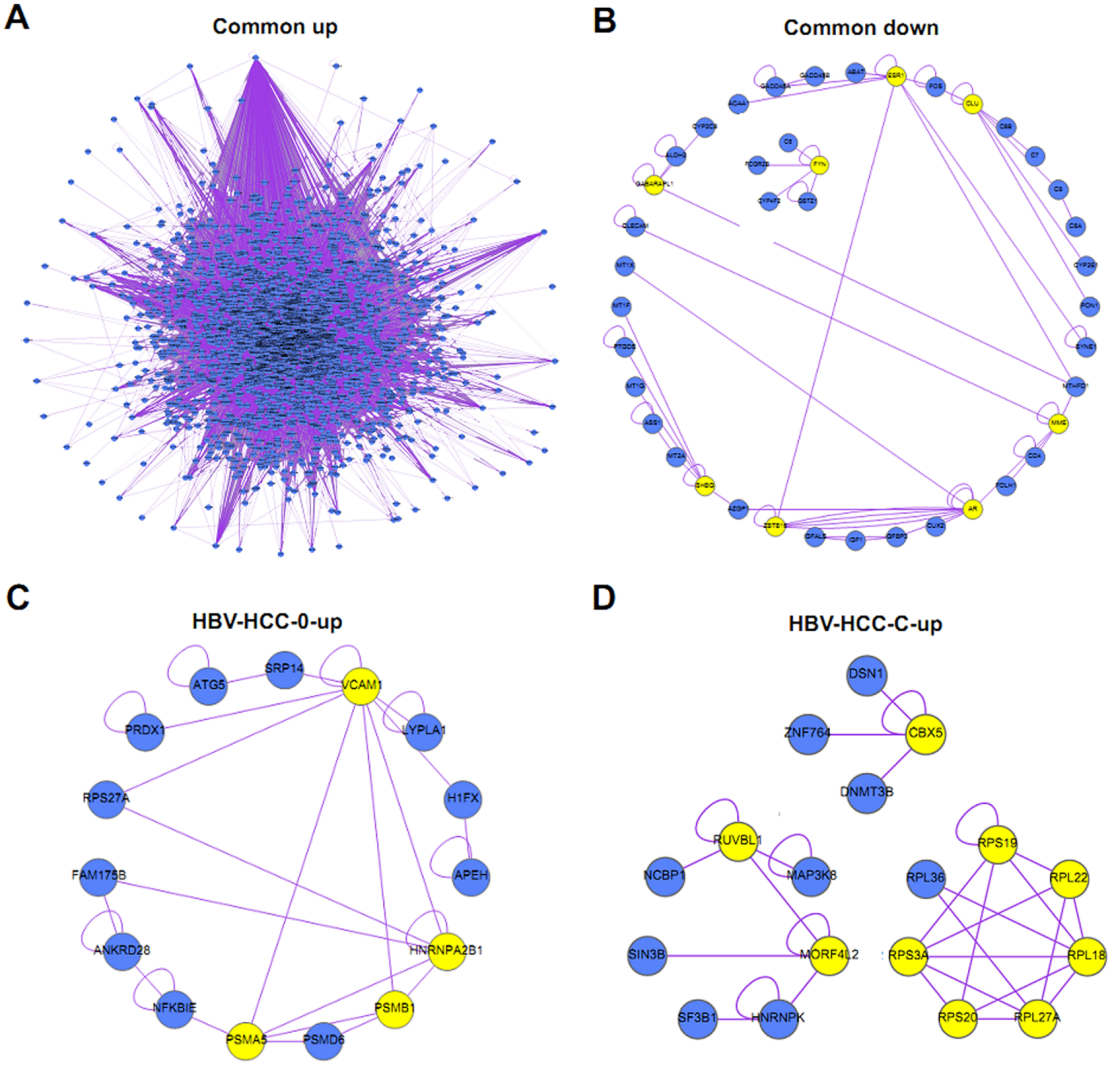
The interactome networks of common and stage-specific DEGsin HBV- related HCC. (A) The interactome networks of upregulated common DEGs. (B)The interactome networks of downregulated common DEGs. (C) The interactome networks of upregulated unique DEGs in BCLC stage 0. (D) The interactome networks of upregulated unique DEGs in BCLC stage C. The yellow nodes in the interactome networks represent significant hub genes.

In order to better understand the interaction of DEGs, we constructed interactome networks of common DEGs using the NetVenn online tool. As Fig 6A shows, the upregulated common DEGs constituted a large and complex network. A relatively small number of downregulated common DEGs formed two networks, and the ESR1, CLU, MME, AR, ZBTB16, SHBG, GABARAPL1, and FYN genes were significant hub genes, interacting with at least three other genes in the interactome networks. As for the stage-specific DEGs, networks could only be constructed with upregulated unique DEGs in BCLC stage 0 and upregulated unique DEGs in BCLC stage C. As shown in Fig 6C, the VCAM1, HNRNPA2B1, PSMB1, and PSMA5 genes were significant hub genes in the interactome networks of DEGs in BCLC stage 0, and CBX5, RUVBL1, MORF4L2, RPS19, RPL22, RPL18, RPL27A, RPS20, and RPS3A were significant hub genes in the interactome networks of DEGs in BCLC stage C of HBV-related HCC.

## Discussion

Treatment strategies for HCC are mainly based on the stage of the disease, and the BCLC staging system is used by most clinicians. However, the molecular characteristics and the associated molecular functions, pathways, and interactions associated with HBV-related HCC in different BCLC stages are not well understood. In the present study, we found that HBV-related HCC in different BCLC stages were not only associated with common DEGs, but also included stage-specific DEGs. These common and stage-specific DEGs were distributed among various chromosomes, and had different molecular functions and different associated biological pathways. In addition, these common and unique DEGs formed complex interactome networks.

Microarrays have been utilized to detect DEGs and non-coding RNA, such as microRNAs and long non-coding RNA in HCC, and especially in HBV-related HCC. Many DEGs have been identified as prognostic and diagnostic markers for the development of HCC [9–11, 20–23]. In routine clinical practice, clinicians use staging systems to design different treatment programs, and the BCLC staging system is the most commonly used system for HCC management [4]. The molecular characteristics of HCC indifferent BCLC stages have not been well explored. In the present study, we reanalyzed microarray data (GSE14520), which was originally published by Roessler S et al [15, 16]. Using GSE14520 microarray expression profile, Roessler S et al. has identified a metastasis gene signature that could as a predictor of overall and disease-free survival of HCC patients that independent on clinical characteristics[15]. In addition, Roessler S et al. investigated the genes whose copy numbers were related to gene expression as well as the progression of HCC, by combining data of GSE14520 with GSE14322 microarray expression profiles [16]. For our present study, we found many common and unique DEGs in HBV-related HCC associated with different BCLC stages. Common DEGs in HCC were found in all BCLC stages, suggesting that these genes are essential factors throughout HCC development, and some of these common DEGs should be considered as potential targets for HCC treatment. In contrast, because the stage-specific DEGs were closely related to specific BCLC stages of HCC, further exploration to determine whether these stage-specific DEGs can be used as biomarkers to optimize the BCLC staging is warranted.

In order to determine whether the common and stage-specific DEGs had distinct distributions in chromosomes, we detected the location of these DEGs in the genomes of HCC cases. We found that a relatively high number of upregulated and downregulated common DEGs were located on chr 1. Furthermore, chr 1 was related to abnormal expression of stage-specific DEGs (either upregulated DEGs or downregulated DEGs) from BCLC stage 0 to C, suggesting that genetic disorders of chr 1 play important roles in the development of HBV-related HCC. In addition, we found that the downregulated unique DEGs in BCLC stage A were located mainly on chr 11, 16, 3, and 4, whereas the upregulated or downregulated DEGs in BCLC stage B were distributed mainly in chr 2 or chr 8, and the upregulated unique DEGs in BCLC stage C were located mainly on chr 19. These results indicated that distinct chromosomes associatedwith dysregulated DEGs were related to specific BCLC stages of HCC. HCC is a disease associated with genetic variants and genomic abnormalities, including mutations, insertions, deletions, and amplifications. Deletions and amplifications of different genes identified by copy number variation (CNV) analysis have been reported in HCC cases, and the most common CNVs in HCC include gains in chr1, 6, 8, 11, 17, 20, and others, and losses in chr 1, 4, 8, 9,13, 14, 16, 17, and others [24–26]. The upregulated and downregulated common and stage-specific DEGs may be associated with CNVs in various chromosomes at different BCLC stages. In addition, the most likely cause of copy number variation of common and stage‑specific DEGs in different chromosomes is HBV integration. During HBV chronic infection, the integration of HBV DNA into host cellular DNA could mediate chromosomal instability with genomic gains or losses that result in copy number variation, and cause the disruption or promotion of cellular gene expression [27]. HBV integration is considered to be a common phenomenon, and localizes on almost all chromosomes as a random event [28].The characteristics of HBV integration may be associated with the distribution of common DEGs that spread throughout the genome. Furthermore, current reports indicated that the hotspots of HBV integration between early onset and late onset disease were rather different [29]. The diverse hotspots of HBV integration in different progress periods, maybe contribute to the distribution of stage-specific DEGs in the chromosomes of HBV-related HCC with different BCLC staging.

The development of HCC is related to alterations in molecular functions and biological pathways [30, 31]. In order to explore whether distinct molecular functions and biological pathways were enriched in different BCLC stages of HBV-related HCC, GO and KEGG analyses were performed to detect common and stage-specific DEGs. In the present study, multiple GO terms and KEGG pathways were found in common DEGs, suggesting that a variety of molecular functions and pathways are involved in the development of HCC. In addition, non-membrane-bound organelles, binding, and lumens were found associated with molecular functions of common DEGs, and some of these biological functions are consistent with abnormalities described in previous reports [32]. The KEGG pathway analysis showed that the upregulated common DEGs participated in pathways of cancer, the cell cycle, and spliceosomes, suggesting these biological functions were critical in development of HCC, whereas the downregulated common DEGs were involved in the metabolism of retinol, drugs, tryptophan, and fatty acids, which is consistent with previous research [11, 33, 34]. The distinct enriched GO terms and KEGG pathways of stage-specific DGEs suggest that specific molecular functions and pathways participate in different stages of HBV-related HCC. Targets for these specific molecular functions and pathways may suggest ways to develop new or improved drugs for HCC in specific stages.

It is widely accepted that HCC is a heterogeneous disease governed by multiple genetic and epigenetic changes that resulted in the deregulation of distinct molecular function and signalling pathways. In HBV-related HCC patients, HBV contributes to the dysregulation of molecular function and signalling pathways with various mechanisms [35–37]. On the one hand, HBV DNA could integrate into the host chromosome, induce genomic instability or direct insertional mutagenesis that result in the progressive accumulation of genomic alterations, which further lead to the progressive deregulation of molecular function and signalling pathways. On the other hand, the viral proteins HBx and/or preS/S could directly alter the activation of diverse molecular function and signalling pathways. In addition, epigenetic regulation of tumor gene expression mediated by viral proteins such as HBx, also are implicated in the abnormality of distinct molecular function and signalling pathways.

In present study, we found that four genes, including JUN (the oncogene encoding c-Jun protein), GADD45G, NR4A1 and DUSP6,which associated with MAPK signaling pathways, were downregulated in patients with HBV-related HCC tissues with BCLC 0 staging than in non-tumor tissues via analysis of DAVID online tools (Fig 5A and S5 Table).Previous study from Endo M et al. showed that no copy number gain of JUN was seen in primary HCC tumors. But compared with non-tumor tissues, a loss of JUN was observed in 38% HCC tumors and expression of JUN was significantly lower in 70% HCC tissues [38], and these data are consistent with our results. Though higher expression levels of c-jun gene in non-tumor tissues are observed, it does not mean that the phosphorylation levels of c-jun are higher in non-tumor tissues than in HBV-related tumor tissues. However, the function of c-jun mainly depends on the phosphorylation state mediated by MAPK pathways [39]. In addition, we found that the rest of 3 genes did not participate in MAPK signalling pathways, but were mainly as regulator (GADD45G) [40] or negative regulator (NR4A1, DUSP6) [41, 42] of MAPK signaling pathways. We support that HBV could activate MAPK signaling pathways through HBx as shown by published reports[43–45], while the role of HBV infection on regulation of different regulators or negative regulators of MAPK pathways should be further investigated in future studies.

In current researches, different gene expression signatures as molecular markers of HCC had been assessed, based on different predictive models, such as random survival forests (RSF) [46],Support Vector Machines (SVM), Nearest Centroid (NC), 3-NearestNeighbor (3-NN) and et al, which mentioned by Roessler et al [15]. The reported gene expression signatures were mainly independent on BCLC staging but related to clinical outcomes such as survival, metastasis and recurrence[12–14]. In addition, current reports suggest that the distinct gene expression signatures could potentially distinguish tumor molecular subtypes, assist to predict clinical outcomes, and provide novel insights for more precise understanding of disease mechanism to design molecular targeted drugs. Based on GSE14520 microarray expression profile, Roessler S et al. have discovered 161 distinct gene expression signatures that could be used as predictor for metastasis[15]. In our study, the identified distinct gene expression signatures combined common DEGs with stage-specific DEGs underlie the molecular basis of the current BCLC staging. Our work provides an apparent link between molecular events with tumor stages but not directly with clincial outcomes that inculded survival, metastasis and recurrence. In addition, the altered expression of DEGs also provides functional insights into BCLC staging-associated features of tumor progress. We support that integration of distinct molecular features of BCLC staging with molecular markers that associated with clinical outcomes via suitable methods is crucial for HCC management, especially in personalized therapy for patients in different BCLC staging with diverse gene expression profiles.

Finally, we investigated the interaction of DEGs, and a large and complex interactome network was constructed from upregulated common DEGs, suggesting complex links among different common DEGs. In addition, several downregulated common DEGs formed two networks, and ESR1, CLU, MME, and other genes were found to be significant core genes in the interactome network. These genes shouldbe considered potential therapeutic targets for HCC in future research.In addition, in BCLC stage 0, VCAM1, HNRNPA2B1, PSMB1, and PSMA5 genes were significant hub genes in theinteractome network, and CBX5, RUVBL1, MORF4L2, and other genes were found to be core genes in the interactome network of unique DEGs in BCLC stage C. These hub genes in BCLC stages 0 and C might be used as biomarkers that complement theBCLC staging system.

In conclusion, usingmicroarray data from HBV-related HCC cases in BCLC stages 0 (very early stage) to C (advanced stage) in the GEO database, many DEGs, including common DEGs and stage-specific DEGs, were identified. In addition, common and stage-specific DEGs showed distinct molecular functions, were involved in various pathways, and formed complex interactome networks. In addition, in the study, our results showed that BCLC staging is an important prognostic factor of survival and recurrence in patients with HBV-related HCC, but current studies showed that BCLC staging often fails to predict the clinical course of high-risk patients [13], because of the molecular heterogeneity of HCC tissues in the same BCLC staging. These suggest that the molecular classification based on BCLC staging is not enough to predict the progression of HCC. Therefore, building comprehensive staging system with integration of molecular classification, based on different gene signatures or pathway signatures that related to diverse clinical outcomes with distinct molecular features of BCLC stages, will provide new insight on HCC development with molecular heterogeneity, predict different outcomes in patient with similar clinical features, and give a guide for personalized medicine therapy by combined traditional treatment with targeting the dominant gene signatures or pathway signatures.

## Supporting Information

S1 Table Characteristics of patients enrolled in this study. (XLS)

S2 Table DEGs in different BCLC stages of HBV-related HCC compared to non-HCC. (XLSX)

S3 Table Detailed information of common DEGs and stage-specific DEGs. (XLSX)

S4 Table GO terms of common DEGs and stage-specific DEGs. (XLSX)

S5 Table KEGG pathways of common DEGs and stage-specific DEGs. (XLSX)
